# Supplementary material for: Dynamic ordering transitions in charged solid
Source: Fundam Res. 2021 Aug 8;2(2):178–83. doi: 10.1016/j.fmre.2021.07.006 (PMC11197670; doi:10.1016/j.fmre.2021.07.006)
Supplement: Supplementary file 1 [file mmc1.docx]

**Supplemental Material for**

**Dynamic ordering transitions in charged solid**

Jian Sun^a,1^, Jiasen Niu ^a,1^, Yifan Li^a^, Yang Liu^a^, L. N. Pfeiffer^b^, K. W. West^b^, Pengjie Wang^c,^*, Xi Lin^a,d,e^*

^a^ International Center for Quantum Materials, Peking University, Beijing 100871, China

^b^ Department of Electrical Engineering, Princeton University, Princeton, New Jersey 08544, USA

^c^ Department of Physics, Princeton University, Princeton, New Jersey 08544, USA

^d^ Beijing Academy of Quantum Information Sciences, Beijing 100193, China

^e^ CAS Center for Excellence in Topological Quantum Computation, University of Chinese Academy of Sciences, Beijing 100190, China

*Corresponding authors.

Email address: xilin@pku.edu.cn (X. Lin); pengjie.wang@princeton.edu (P. Wang)

^1^These authors contributed equally to this work.

**Contents:**

1. Sample geometry and noise spectrum measurement setup

2. Transport behavior of the bubble states and the FQH states

3. Noise spectra for other states

4. Additional experimental results for the R2c state

5. Critical exponent

6. Time-domain signals

7. Electron temperature

**1. Sample geometry and noise spectrum measurement setup**

In a commonly used van der Pauw [1] or Hall-bar [2] shaped 2DEG sample, electron solids form in the bulk, accompanied by quantized edge conductions [3-5]. Therefore, the measured electrical signals contain the information from both the edges and the bulk of the sample. Our sample was fabricated into Corbino geometry [6] (see the sketch in Fig. S1), so only the properties of electron solids were probed without the influence of edge states.

When performing the noise spectrum measurement, the noise and the conductance were measured at the same time. Figure S1 shows the schematic of the measurement setup.

**Fig. S1.** Schematic of the noise spectrum measurement setup. At room temperature, a waveform generator (Agilent 33220A) and a divider were used to apply dc and ac voltages between inner and outer contacts. In the dilution refrigerator, Thermocoax filters [7] and cryogenic RC filters [8] were used to reduce the electron temperature of the sample. The cut-off frequency of the filtering combination was about 16 kHz, which does not influence the noise spectrum measurement. The noise and conductance signals were both amplified by a low noise amplifier (NF-CA5350, gain 1MV/A) outside the cryostat at room temperature. The noise signals were digitized by a data acquisition (DAQ) card (National Instrument USB-6289, 18 bits, sampling rate 600,000 Hz), and the conductance signals were recorded by a lock-in amplifier.

**2. Transport behavior of the bubble states and the FQH states**

Figure S2a-c are depinning traces of the bubble states R2a, R2b and R2d, respectively. Using the same separation criteria as those used in the R2c state, all these traces can also be divided into three distinct regions, A, B, and C, by *E*_t_ and *E*_d_ (dashed black vertical lines), respectively. Figure S2d-f are the breakdown traces of the FQH states at *ν* = 8/3, *ν* = 5/2 and *ν* = 7/3, respectively. Comparing the trace features of the bubble states and the FQH states, we find that the former ones fluctuate more violently and have pronounced hysteresis from opposite sweeping directions.

**Fig. S2.** Conductance *G* vs. electric field *E* for different states at 12 mK. (a-c) Depinning traces of the bubble states R2a, R2b and R2d, respectively. The dashed black vertical lines are used to separate the traces into three regions: A, B and C. (d-e) Breakdown traces of the FQH states at *ν* = 8/3, *ν* = 5/2 and *ν* = 7/3, respectively. The *E* sweeping directions are noted as following: negative to positive (solid blue line) and positive to negative (dashed yellow line), as shown in each figure.

**3. Noise spectra for other states**

The dynamic ordering alternation behavior can also be observed in other bubble states, for example, in the R2d state as shown in Fig. S3. The dashed vertical line is a guide line showing where the noise signals disappear, and it is also close to the last obvious conductance dip as shown in Fig. S3a. For comparison, the same measurements were taken in the quantum Hall states, such as in the *ν* = 3 IQH state and *ν* = 5/2 FQH state (Fig. S4). The results show that no noise signal appears. As for the bubble states in higher Landau levels (*ν* > 4), noise spectrum measurements have been carried out in previous works [9, 10], and signs of dynamic ordering alternations can be found in the R4a state from the reference [9].

Fig. S3. Conductance and noise spectra for the R2d state at 12 mK. (a) Conductance *G* as a function of electric field *E*. (b) Noise spectral density *S*_I_ as a function of frequency *f* and electric field *E*. The dashed black vertical line is a guide line showing where the noise signals disappear, which is close to the field of the conductance dip. Both Fig. S3a and S3b were measured at *B* = 4.62 T, slightly deviating from the center of the R2d state (4.60 T), and hence the conductance at *E* = 0 V/m has a finite value.

Fig. S4. Conductance and noise spectra of the *ν* = 3 IQH state and *ν* = 5/2 FQH state at 12 mK. There is no narrow-band or broad-band noise in both the *ν* = 3 IQH state and *ν* = 5/2 FQH state.

**4.** **Additional experimental results for the R2c state**

To verify that the noise signals do not appear when *E* > *E*_d_ (11.30 V/m), here we present more experimental results for the R2c state at 12 mK with *E* up to 22.00 V/m (Fig. S5). The results show that narrow-band noise and broad-band noise no longer appear when *E* is larger than *E*_d_. Moreover, the temperature dependence of the noise spectra was also measured, and the results are shown in Fig. S6. Similar to the case at 12 mK, both narrow-band noise and broad-band noise disappear when *E* is larger than the *E*_d_ at 35 mK (Fig. S6a). At 45 mK and a higher temperature, 100 mK, no noise signal appears (Fig. S6b & S6c).

Fig. S5. Conductance and noise spectra for the R2c state. (a) Conductance *G* as a function of electric field *E* at 12 mK. The dashed red line is the cubic polynomial fitting of the conductance data to determine the value of *E*_d_ = 11.30 V/m. (b) Noise spectral density *S*_I_ as a function of frequency *f* and electric field *E* at 12 mK. The dashed black vertical line is a guide line showing where the noise signals disappear, which is near the dip of the dashed red line. There is no noise signal when *E* > *E*_d_.

Fig. S6. Temperature dependence of the conductance and noise spectra for the R2c state. (a) Conductance *G* and noise spectra measured simultaneously as a function of *E* at 35 mK. The dashed black vertical line is a guide line showing where the noise signals disappear, which is near *E*_d_. (b, c) Conductance *G* and noise spectra measured simultaneously as a function of *E* at 45 mK and 100 mK respectively, and no noise signal appears at these two temperatures.

Fig. S7. Noise spectra for the R2c state at 12 mK before instrumental noise was subtracted. The horizontal lines running across the entire image are artificial signals from the measurement setup.

Fig. S8. Noise floor. The solid red line is the noise spectrum measured at *E* = 0 V/m, where the electron solid remains pinned, and thus no noise signal from the dynamics is expected to exist. All the sharp peaks are artificial signals from the measurement setup at fixed frequencies, which correspond to the horizontal lines in Fig. S7. The dashed blue line is a guide line indicating the noise floor.

**5. Critical exponent**

In the main text, critical exponent *β* at 35 mK has been calculated by choosing the same *E*_t_ (1.30 V/m) as the value used at 12 mK. The results show that *β* at 35 mK is larger than that at 12 mK, and both are larger than 1. The existence of conductive electron liquid under zero bias at 35 mK prevents us to extract the actual value of *E*_t_ from the conductance trace (Fig. S6a) directly. Moreover, considering that the thermal motion of electron solids becomes more pronounced at elevated temperatures, the pinned electron solids are easier to be depinned, and thus the actual value of *E*_t_ may be smaller than 1.30 V/m. To figure out how *β* changes as *E*_t_ decreases at 35 mK, here we choose *E*_t_ = 0 V/m as an example (Fig. S9). The results show that *β* at 35 mK increases from 3.29 ± 0.03 to 4.04 ± 0.04 by decreasing *E*_t_ from 1.30 V/m to 0 V/m, and thus the conclusion that *β* at 35 mK is larger than that at 12 mK (2.63 ± 0.07) remains unchanged.

Fig. S9. Slope of the narrow-band noise stripes at 35 mK if choosing *E*_t_ = 0 V/m. The calculated critical exponent *β* changes to 4.04 ± 0.04 if choosing *E*_t_ = 0 V/m.

**6. Time domain signals**

Figure S10a & S10b show the time domain signals corresponding to the spectrum dominated by narrow-band noise (Fig. 2a) and broad-band noise (Fig. 2b) in the main text, respectively. The oscillations of the current background are coming from the external 17 Hz ac excitation. In Fig. S10b, besides the current oscillations, there are also interruptions marked by dashed red circles which contribute to the generation of the broad-band noise in the noise spectrum. The interruptions seem to occur at the bottom of the current background oscillation. To find out whether the generation of the interruptions is related to the 17 Hz ac excitation, we carried out the same measurement without 17 Hz ac excitation. The results show that the interruptions still exist (dashed red circles in Fig. S10c). Therefore, we demonstrate that the generation of the broad-band noise is independent of the 17 Hz ac excitation. The time domain signals in Fig. S10a-c are all digitally filtered (low-pass, from 0 to 5 kHz) to eliminate the influence of high frequency irrelevant noise.

**Fig. S10.** Time domain signal examples for the R2c state. (a) Time dependence of the current signal corresponds to the spectrum dominated by narrow-band noise as shown in Fig. 2a. Evenly-spaced sharp current peaks are the narrow-band noise signals, while the oscillations of the current background are coming from the external 17 Hz ac excitation. The inset shows a zoom-in graph for better clarity of the periodic oscillations, where the evenly-spaced dashed red lines are used for eye-guide. (b) Time dependence of the current signal corresponds to the spectrum dominated by broad-band noise as shown in Fig. 2b. There are several places where oscillations are interrupted (dashed red circles), which generates the broad-band signal in the noise spectrum. (c) Time domain signals without 17 Hz ac excitation were measured in order to exclude the correlation between the interruptions and the 17 Hz ac excitation.

**7. Electron temperature**

The temperature mentioned in the main text is the electron temperature, which is equal to the refrigerator temperatures in this work. The electron temperature is confirmed by the temperature dependence of the *ν* = 14/5 FQH state’s conductance as shown in Fig. S11.

Fig. S11. 12 mK electron temperature. (a) Temperature dependence of the conductance in the lower spin branch of the second Landau level (2 < *ν* < 3). (b) Arrhenius plot of the 14/5 FQH state (the dip of the grey-shaded region in Fig. S11a). The linear relation agrees with that the electron temperature is equal to the refrigerator temperature at least above 12 mK.

**Supplemental material reference**

[1] J. Gores, G. Gamez, J. H. Smet, et al., Current-induced anisotropy and reordering of the electron liquid-crystal phases in a two-dimensional electron system. Phys. Rev. Lett. 99 (2007) 246402.

[2] H. Fu, P. Wang, P. Shan, et al., Competing ν = 5/2 fractional quantum hall states in confined geometry. Proc. Natl. Acad. Sci. USA 113 (2016) 12386-12390.

[3] J. Eisenstein, K. Cooper, L. Pfeiffer, Insulating and fractional quantum hall states in the first excited landau level. Phys. Rev. Lett. 88 (2002) 076801.

[4] K. Cooper, M. Lilly, J. Eisenstein, et al., Insulating phases of two-dimensional electrons in high landau levels: Observation of sharp thresholds to conduction. Phys. Rev. B 60 (1999) R11285-11288.

[5] R. Du, D. Tsui, H. Stormer, et al., Strongly anisotropic transport in higher two-dimensional landau levels. Solid State Commun. 109 (1999) 389-394.

[6] X. Wang, H. Fu, L. Du, et al., Depinning transition of bubble phases in a high landau level. Phys. Rev. B 91 (2015) 115301.

[7] A. B. Zorin, The thermocoax cable as the microwave frequency filter for single electron circuits. Rev. Sci. Instrum. 66 (1995) 4296.

[8] P. Wang, K. Huang, J. Sun, et al., Piezo-driven sample rotation system with ultra-low electron temperature. Rev. Sci. Instrum. 90 (2019) 023905.

[9] K. B. Cooper, J. P. Eisenstein, L. N. Pfeiffer, et al., Observation of narrow-band noise accompanying the breakdown of insulating states in high landau levels. Phys. Rev. Lett. 90 (2003) 226803.

[10] K. Bennaceur, C. Lupien, B. Reulet, et al., Competing charge density waves probed by nonlinear transport and noise in the second and third landau levels. Phys. Rev. Lett. 120 (2018) 136801.
